# A narrative literature review of palliative care regarding patients with idiopathic pulmonary fibrosis

**DOI:** 10.1002/nop2.163

**Published:** 2018-06-03

**Authors:** Yasuko Igai

**Affiliations:** ^1^ St. Luke's International University Tokyo Japan

**Keywords:** advanced care planning, chronic respiratory disease, end‐of‐life care, idiopathic pulmonary fibrosis, interstitial lung disease, narrative literature review, nursing, palliative care

## Abstract

**Aim:**

The aim of this study was to examine the reported characteristics of extant studies on palliative care for patients with idiopathic pulmonary fibrosis.

**Design:**

Narrative review.

**Methods:**

A comprehensive search of the following electronic databases in English and Japanese commenced from 2002 ‐ December 2017. Eligibility criteria was determined by the inclusion and exclusion criteria.

**Results:**

Nineteen articles were eligible. The characteristics of palliative care for patients with idiopathic pulmonary fibrosis were symptoms relief, start time of palliative care and palliative care needs of patients and care partners. Also, patients' education of disease management including advanced care planning and developing a palliative care system by the healthcare provider including multidisciplinary professional teams was identified. The care provided was a “care conference” and integrated palliative care was carried out in the patient's home. The majority of the studies were qualitative and retrospective in design. The palliative care system and the development of palliative care were limited.

## 1 INTRODUCTION

Idiopathic pulmonary fibrosis (IPF) is a refractory and progressively fatal disease. The course is variable and unpredictable; patients can have long periods of stability and then have sudden debilitating episodes (Raghu et al., 2011). Some patients experience the episodes of acute respiratory deterioration. There are also cases with a shorter life expectancy than with other malignant diseases and acute exacerbations of unknown cause may lead to a life‐threatening crisis (Raghu et al., 2011). Evidence‐based therapies and diagnostic procedures have not been fully established (Purokivi, Hodgson, Myllarniemi, Salomaa, & Kaarteenaho, 2017), but may be indicated for lung transplantation. In addition, antifibrotic drugs such as nintedanib and pirfenidone do not provide an improvement in life prognosis (Canestaro, Forrester, Raghu, Ho, & Devine, 2016).

Optimal medical treatment for IPF has yet to be found. The sensation of dyspnoea is one of the characteristic symptoms of IPF, which impedes the patient's independent living capacity. Moreover, research found that those patients' experiences of coping with dyspnoea brought up questions and concerns about the meaning of their illness experiences (Igai, 2016). Therefore, researchers began urging that specific goals fall within the realm of palliative care and needed to include a focus on symptom control (Gilbert & Smith, 2009). The goal of palliative care is improving the quality of life (QOL). Health‐related QOL of patients with IPF tends to be low, particularly in the domain of physical and psychological well‐being (de Vries, Kessels, & Drent, 2001; Tomioka, Imanaka, Hashimoto, & Iwasaki, 2007). Dyspnoea was found to be the main factor that caused a decline in the quality of life for patients with IPF (Nishiyama et al., 2005).

The IPF statement (Raghu et al., 2011) noted that patients and families also sought symptomatic relief from phenomenon such as physical, emotional and psychological distress and needed spiritual support. Lee, McLaughlin, and Collard (2011) concluded from their systematic review of the IPF research that the focus on care for patients with IPF should include disease‐centred management, symptom management and palliative care integrated in routine care, which includes advanced care planning (ACP). The NICE Clinical Guidelines in UK (2013) provided evidence‐based results indicating that variations existed in the components of best supportive care. It stated the necessity of (a) symptomatic relief; (b) oxygen therapy; (c) psychological support for conditions such as depression and anxiety; and (d) spiritual support for questions such as the meaning and purpose of life.

Patients with cancer tended to receive palliative care for the full range of painful situations including spiritual pain when searching for meaning of life and purpose; unfortunately patients with IPF are still suffering from the burden of symptoms and emotions (Igai, 2016). In the systematic review on nursing to improve the quality of life for IPF patients, much of the research was qualitative and retrospective research. For this reason, the author adopted the narrative literature review method and investigated palliative care aimed at improving the quality of life for patients with IPF.

## 2 AIMS

The aim of this narrative literature review was to examine the characteristics of extant studies on palliative care for patients with IPF.

## 3 METHODS

### 3.1 Ethical considerations

Study participants from reviewed studies had been provided informed consent. The patients from case reports and retrospective studies had an informed consent waiver not an actual informed consent. Research included in this narrative review had received ethical approval.

### 3.2 Methodological approach

This narrative review was based on the methods of Cipriani and Geddes (2003) and Pae (2015). Accordingly, the research method (Cipriani & Geddes, 2003; Pae, 2015) did not have a predefined protocol‐basis and the hypothesis was a broad overview of the topic‐related research area. The search method necessarily involved subjective selection bias, with the search media mainly from PubMed or Medline database. The data extraction was a simple description of study findings and interpretation containing the authors' subjective intention.

The author conducted the comprehensive search of electronic databases in English and Japanese, which included the following: the Cochrane Central Register Controlled Trials (CENTRAL), CINAHL with the full text, MEDLINE, PsycINFO, Embase and Ichushi‐Web (Japan Medical Abstracts Society database). The review was limited to studies published between 2002, which was when the WHO defined palliative care to 2017. The following combination of MESH and keywords was used: [interstitial pulmonary fibrosis], [idiopathic pulmonary fibrosis] AND [palliative care]. This narrative review search string was accessed 9 December 2017.

### 3.3 Eligibility criteria

Eligibility criteria included research of mainly patients with IPF and in English or Japanese. Exclusion criteria included only drug or treatment interventions, studies of medical economics, reviews, letters to editor and commentaries, animal studies, basic science research and duplicates.

### 3.4 Research screening method

For the retrieved documents, one reviewer provided the primary screening to satisfy the eligibility criteria based on the article title and abstract. Research was not included if it did not clearly meet the eligibility criteria. In the secondary screening, the same reviewer carefully read the full article and using a matrix documented the researcher's discipline, country, research design, aim, sample, age, gender, environment, the content of the intervention and outcomes. No further articles were excluded during the phase of a full article review.

## 4 RESULTS

### 4.1 Search results

**Figure 1:**
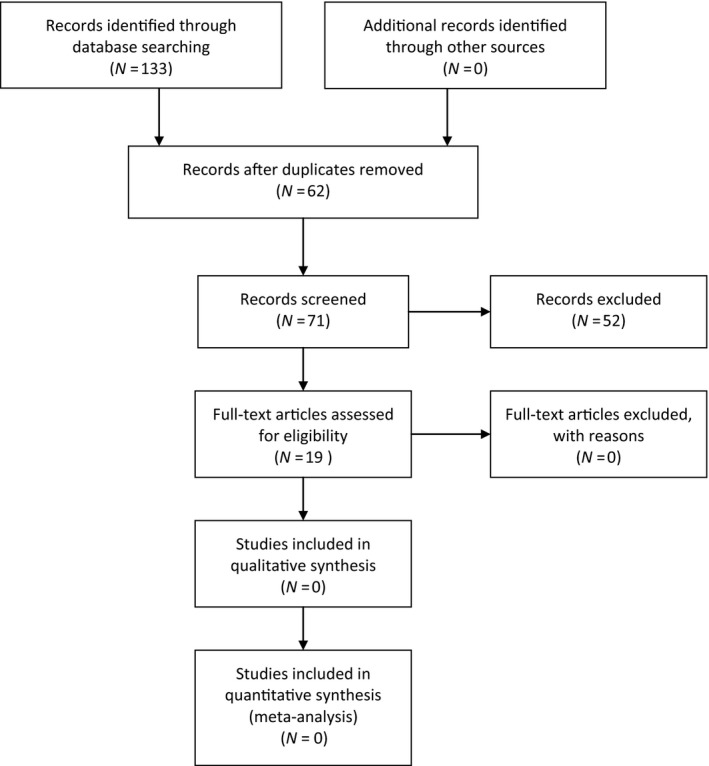
Study flow diagram literature searches

By applying the described strategy, 133 articles were found, 67 of which were relevant hits. After carefully sorting through the relevant hits, the duplicates were removed. Applying the exclusion criteria, 19 articles were found appropriate and included (Figure 1).

### 4.2 Characteristics of the existing studies on palliative care

Characteristics of the existing studies are summarized in Table 1. Countries where the studies were conducted were the UK, (Bajwah et al., 2013, 2015; Duck et al., 2015; Sampson, Gill, Harrison, Nelson, & Byrne, 2015; Sharp et al., 2017), the USA (Liang et al., 2017; Lindell, Kavalieratos, Gibson, Tycon, & Rosenzweig, 2017; Lindell et al., 2010, 2015; Rush, Berger, & Celi, 2017; Swetz, Carey, & Bundrick, 2012), Canada (Kalluri, Claveria, Haggag, & Richman‐Eisenstat, 2016; Kalluri et al., 2017, 2017; Periyakoil, Skultety, & Sheikh, 2005), Japan (Arita et al., 2010), Finland (Rajala et al., 2016), Denmark (Overgaard et al., 2016) and Sweden (Ahmadi et al., 2016). The combined 19 studies comprised 1708 patients and 74 care partners. The age range was between 52 and 78 years. The environments where patients received palliative care were home care (Arita et al., 2010; Bajwah et al., 2013; Duck et al., 2015; Kalluri et al., 2017; Lindell et al., 2010, 2017; Overgaard et al., 2016; Periyakoil et al., 2005; Rajala et al., 2016; Sampson et al., 2015; Sharp et al., 2017; Swetz et al., 2012), inpatient ward (Arita et al., 2010; Rush et al., 2017), intensive care unit (Rajala et al., 2016), hospice care (Liang et al., 2017; Lindell et al., 2015) and outpatient respiratory centres (Ahmadi et al., 2016; Bajwah et al., 2015).

**Table 1:** Main results of extant research on palliative care for patients with IPF by study design

Authors and Year	Main Results
Mixed methods study	
Bajwah et al. (2015). (UK)	Mixed methods study (*N* = 25; 67 SD 11 years) of the feasibility and acceptability on the impact of a case conference intervention delivered in the home showing that community case conferences improved the quality of life.
Lindell et al. (2010). (US)	Mixed methods study (*N* = 21; 66 SD 11 years) of the disease management programme to decrease symptom burden and stress and improve the HRQOL finding that intervention group rated their HRQOL less positively.
Quantitative study	
Ahmadi et al. (2016). (Sweden)	A national wide registry‐based cohort study between 2011 and 2013 (*N* = 285; 78 SD 8 years) comparing the palliative care between dying patients with ILD and LC showing that ILD patients received poorer access.
Arita et al. (2010). (Japan)	Historical cohort study between 1989 ‐ 2008 (*N* = 57; 73^a^ years) grasping the future points to be considered in palliative care showing that palliative care was limited the terminal stage patients.
Kalluri et al. (2016). (Canada)	Historical cohort study between 2009 and 2016 (*N* = 67) determining the impact of our MDC ILD Clinic showing that the group receiving care by the MDC had no ER visits for dyspnoea.
Kalluri et al. (2017). (Canada)	Historical cohort study between 2009 and 2012 (*N* = 32) exploring the acute care utilization in the last year of life and location of death by MDC showing that the group receiving care by the MDC had reduced health care use and more home deaths.
Liang et al. (2017). (US)	Historical cohort study between 2001 and 2012 (*N* = 106; 60^a^ years) of the describing characteristics of IPF patients centre showing that mortality associated with ICU admission, patients and families should be informed about PC early following diagnosis of IPF.
Lindell et al. (2015). (US)	Historical cohort study (72 SD 9 years) of the timing of referral for palliative care showing that only 38 (13.7%) had a formal palliative care referral and the majority (71%) was referred within 1 month of their death.
Rajala et al. (2016). (Finland)	A national prospective IPF cohort study (*N* = 59; age 77^a^ years) of treatment practices, decision‐making and symptoms showing that the frequent opioids use is for relieving symptoms, with late end‐of‐life decisions.
Rush et al. (2017). (US)	Historical cohort study using hospital admissions between 2006 ‐ 2012 (*N* = 3166) of patients undergoing mechanical ventilation with 408 patients (12.9%) encountering palliative care (PC) and PC use had increased almost 10‐fold between 2006 and 2012.
Sharp et al. (2017). (UK)	Historical cohort study between 2010 ‐ 2015 (*N* = 73; 49.3%) using a supportive care decision aid tool showing that the documented discussion of referral to palliative care and end‐of‐life discussions yielded significant increases. Palliative care services had not been used in patients with IPF.
Qualitative study	
Bajwah et al. (2013). (UK)	Semistructured in‐depth interviews (*N* = 12; 56–81^b^ years) of the palliative care needs showing the regarding physical and psychosocial needs, impact of disease on social activities were extracted.
Duck et al. (2015). (UK)	Semistructured interviews (*N* = 23; 67^a^ years) of the understanding the perceptions, needs and experiences showing the identified struggling to get a diagnosis and loss of the life were reported.
Kalluri et al. (2017). (Canada)	Case report (*N* = 1; 72 years) of the palliative care providing by the multidisciplinary physicians showing that the first care component was advanced care planning.
Lindell et al. (2017). (US)	Focus group interviews (*N* = 13; 71 SD 8 years) of the perceptions of palliative care needs in patients showing the overwhelming symptom burden and reluctance to engage in advance care planning were extracted.
Overgaard et al. (2016). (Denmark)	Semistructured interviews (*N* = 45; 71^a^ years) of the increasing knowledge of patient's life showing that the sox themes reported the regarding the reactional dyssynchrony and adapted coping strategies.
Periyakoil et al. (2005). (Canada)	Case study (*N* = 3; 77^a^ years) of the control of the dyspnoea using the measurement of the API showing that the effective medical treatment brought the less frequent and less intense panic episodes of dyspnoea.
Sampson et al. (2015). (UK)	Semistructured interviews (*N* = 27; 72^a^ years) of the patient's perspectives showing that the encounters were the identification of changes and the coping strategies in health status.
Swetz et al. (2012). (US)	Case study (*N* = 1; age 58^a^ years) of describing the control of the dyspnoea, showing that the systematic opioids and oxygen therapy use was the mainstay of therapy in the treatment of dyspnoea.

a Median.

b Range; HRQOL, health‐related quality of life; UK; United Kingdom, US; United States.

ILD, interstitial lung disease; LC, lung cancer; MDC, multidisciplinary and collaborative ILD clinic; ER, emergency room; ICU, intensive care unit; API, acute panic inventory.

### 4.3 Types of studies of palliative care for patients with IPF

#### 4.3.1 Mixed methods study

There were two mixed methods study. They both included randomized controlled trials (Bajwah et al., 2015; Lindell et al., 2010).

Bajwah et al. (2015) using mixed methods aimed to obtain preliminary information on the impact of a case conference intervention delivered in the home (Hospital2Home) on palliative care concerns of patients and their care givers and to evaluate programme feasibility and acceptability. The measurement was the palliative care outcome scale (POS). This study reported that community case conferences improved palliative‐targeted symptoms and quality of life after 4 weeks of intervention with patients. The qualitative interview analysis found that nine categories characterized their intervention: support in the community, individual care plans and practical problems addressed, coordination of care and efficiency, crisis management, palliative care, psychological support, symptom control, empowering health professional and advance care planning. This intervention helped patients and care givers manage the uncertainty of illness by facilitating early discussions about disease progression, improving communication and addressing end‐of‐life planning needs.

Lindell et al. (2010) aimed to test the ability of the Program to Reduce Idiopathic Pulmonary Fibrosis Symptoms and Improve Management (PRISIM) intervention to decrease symptom burden and decrease stress. The patient's education included advanced care planning and improving perceptions of Health Related Quality of Life (HRQOL). Quantitative measurements were the University of California San Diego Shortness of Breath Questionnaire (SOBQ), Beck Anxiety Inventory (BAI), Beck Depression Inventory‐II (BDI‐II), Perceived Stress Scale (PSS) and the physical and mental domains of the Short Form (SF)‐36 (Version 2). Lindell et al. (2010) reported that the intervention group rated their HRQOL less positively (*p* = 0.038) and tended to report more anxiety (*p* = 0.077) compared with controls; however, the care partners rated their own stress at a lower level (*p* = 0.018) compared with controls. The themes from the analysis of qualitative data were “did not feel isolated when participating in a disease management program,” “able to put individual disease into perspective,” and “felt that it was important to participate in research to help other with the disease.”

#### 4.3.2 Quantitative studies

##### Retrospective studies

Retrospective studies were nine (Ahmadi et al., 2016; Arita et al., 2010; Kalluri et al., 2016, 2017; Liang et al., 2017; Lindell et al., 2015 Rajala et al., 2016; Rush et al., 2017; Sharp et al., 2017). Ahmadi et al. (2016) reported interstitial lung disease (ILD) was similar to IPF and end‐of‐life discussions with the patients were less common than for those with lung cancer (LC) (41% vs 59%). Moreover, patients with IPF had more unrelieved breathlessness, pain and anxiety compared with patients with LC and received poorer access to specialist end‐of‐life care services.

Arita et al. (2010) studied 20 years (1990–2010) of palliative care research. Palliative care had been limited to patients in the terminal stage and families determined resuscitation when the patient had a sudden negative change of condition. The researchers identified points to be considered for the future in palliative care: advance care planning, so patients' wishes could be honoured when they experienced sudden life‐threatening changes; daily communication between patients and doctors and the construction of a home support system.

Kalluri et al. (2016) reported on the impact of their multidisciplinary and collaborative ILD clinic (MDC), which included a pulmonary rehabilitation/palliative respiratory care specialist and collaboration with the patient's primary care provider (MD or NP) and their provincial home care team including a respiratory therapist. They examined the impact on acute care use (frequency of emergency room visits for dyspnoea and hospitalizations) and place of death of patients with IPF. Of the MDC group, 33% had two or more hospitalizations. There was a significant association between ER visits for dyspnoea (*p* = 0.005) and the number of hospitalizations (*p* = 0.023) in the MDC group, and 78% died at home or in hospice while 22% died in the hospital. In the non‐MDC group, 80% were hospitalized more than twice, 40% had ER visits for dyspnoea, 40% died at home or in hospice and 60% in hospital.

Kalluri et al. (2017) reported on the effectiveness of the multidisciplinary collaborative care (MDC) model to manage patients in their homes and support caregivers. Patients with IPF the receiving MDC care were 24.2 times less likely to have respiratory‐related ER visits (*p *= 0.002) and 2.32 times less likely to have respiratory‐related admissions (*p *= 0.064). Furthermore, patients in MDC care were 9.2 times more likely to die at home or in hospice compared with non‐MDC care (*p *= 0.037). The MDC care model for patients with IPF was thought to reduce the use of health care in the last year of life and increased home deaths.

Lindell et al. (2015) reported that, of the 404 decedents only, 38 (13.7%) had a formal palliative care referral and most (71%) were referred within 1 month of their death. Decedents who died in the academic medical centre ICU were significantly younger than those who died in a community hospital ward (*p *= 0.04) or hospice (*p *= 0.001).

Liang et al. (2017) reviewed records over years from a lung specialty clinic and identified 106 patients with IPF. Only four (3.8%) patients had received a palliative care referral before ICU admission. The majority (77%) died during ICU admission. They suggested that patients and families should be informed about palliative care early following the diagnosis of IPF.

Rajala et al. (2016) reported that the majority of patients with IPF died in a hospital with ongoing life‐prolonging procedures until death. End‐of‐life decisions were still made very late in the illness trajectory. They surmised that an early introduction of integrated palliative care with advance care planning could improve the end‐of‐life care of patients dying from IPF.

Rush et al. (2017) reviewed the hospital admissions of patients with IPF undergoing mechanical ventilation between 2006 and 2012 and their use of palliative care. There were 408 patients (12.9%) who encountered palliative care. The use of palliative care had increased almost 10‐fold between 2006 ‐ 2012.

Sharp et al. (2017) explored the impact of a supportive care decision aid tool using quality improvement methodology. This decision aid tool was assessed with 73 patients with IPF and completed for 49.3% of patients. The documented discussion of referral to palliative care (11.2% vs. 53.6%, *p *< 0.01) and end‐of‐life discussions (15.7% vs. 91.8%, *p *< 0.01) showed significant increases. For patients completing the tool, there was an increase in referrals for palliative care (2.7% vs. 16.7%, *p *< 0.01).

#### 4.3.3 Qualitative studies

##### Semistructured in‐depth interview

There were five semistructured in‐depth interview studies (Bajwah et al., 2013; Duck et al., 2015; Lindell et al., 2017; Overgaard et al., 2016; Sampson et al., 2015).

Bajwah et al. (2013) explored the specialist palliative care needs of people living with end‐stage progressive idiopathic fibrotic interstitial lung disease. Analysis of semistructured in‐depth interview yielded four main themes: the extent of profound physical and psychosocial needs, health‐care professionals' experience of symptom control, impact of disease on social activities and reliance on others and the change in relationships. This study concluded that knowledge and confidence varied among health professionals in managing symptoms and they often underestimated patients' psychosocial needs.

Duck et al. (2015) strove to understand the perceptions, needs and experiences of patients with IPF and found there was an urgent need for a better understanding of the difficulties faced by patients with IPF and their caregivers. Three main themes: “struggling to get a diagnosis”, “loss of the life I previously had” and “living with idiopathic pulmonary fibrosis” were identified.

Lindell et al. (2017) aimed to explore the perceptions of palliative care needs in patients with IPF and their caregivers. Four themes were identified: frustration with the diagnostic process and education received, overwhelming symptom burden, reluctance to engage in advance care planning and comfort in receiving care from pulmonary specialty centre because of resources. Patients and caregivers had a limited understanding of the potential benefits of palliative care.

Overgaard et al. (2016) aimed to increase the knowledge of “living with IPF” for patients and family caregivers. The following six themes emerged: information and disclosure, reactional dyssynchrony, perpetual vigilance, emotional ambivalence, gradual and tacit role shift and adapted coping strategies. Researchers also noted that patients and their caregivers demanded a palliation plan and that greater effort should be made to provide palliative care to patients with IPF starting at the time of diagnosis.

Sampson et al. (2015) explored the perspectives of patients and their caregivers across the IPF disease spectrum. The results would inform the development of clinical pathways and multidisciplinary service interventions. The foci of clinical encounters were: (a) the timely identification of changes in health status, functional activity and understanding of symptoms; (b) medical interventions; (c) coping strategies; and (d) care givers' roles. These researchers also found that patients diagnosed with IPF had little understanding of how their disease would progress and how it would be managed.

#### 4.3.4 Case study

There were three case reports (Kalluri & Richman‐Eisenstat, 2017; Periyakoil et al., 2005; Swetz et al., 2012). Kalluri et al. (2017) reported on “care through collaboration” that was comprised of a registered respiratory therapist, nurse practitioner (NP), palliative care nurse, occupational therapist (OT) and physical therapist (PT) along with the ILD multidisciplinary physicians. The researchers suggested that the first care component of patients with IPF was ACP.

Periyakoil et al. (2005) reported on three case studies involving panic episodes that accompanied dyspnoea. The panic episodes were quantified using the Acute Panic Inventory (API). In one case, the patient experienced less frequent and less intense panic attacks from effective medical treatment and was able to spend time with friends and family.

Swetz et al. (2012) concluded that the use of systematic opioids was appropriate as a mainstay of therapy in the treatment of dyspnoea. Oxygen therapy should also be included although compared with opioids, it had less impact on the distress of dyspnoea.

## 5 DISCUSSION

The studies of palliative care for patients with IPF were limited. Qualitative and retrospective research comprised most of the papers. Palliative care research for patients with IPF were thought to be appropriately situated at the early stage involved in describing the phenomenon of patients' experience. Mixed methods study designs were used to study interventions for palliative care. That approach was adopted to qualify and quantify the impact of the intervention on the patients and to deeply understand the patient's experience.

### 5.1 Characteristics of existing studies in palliative care

Regardless of site where patients received care, palliative care was usually requested. Furthermore, three studies corroborated the recommendations to introduce palliative care at the time of or early after diagnoses (Bajwah et al., 2015; Liang et al., 2017; Lindell et al., 2017).

In qualitative research, it was stated that the patients suffered from dyspnoea and cough, losing autonomy due to the constraints of using home oxygen therapy and losing their ability to enact their usual social roles (Bajwah et al., 2013; Duck et al., 2015; Overgaard et al., 2016). For this reason, patients with IPF were considered to have a unique experience that degraded the quality of their lives. Therefore, health‐care professionals must carefully consider how and when to support the introduction of palliative care that includes introducing how palliative care might improve the patients' quality of life.

In addition, the effectiveness of patients' education consisted of integrated care interventions, patient education by group sessions and respiratory rehabilitation (Bajwah et al., 2015; Kalluri et al., 2016, 2017; Lindell et al., 2010; Rajala et al., 2016). Programmes to support patients with COPD included integrated care models combining drug therapy, oxygen therapy, nutritional advice, self‐management education and psychological support (Nici & ZuWallack, 2012). Support by multiple experts was thought to have provided spiritual wellness through social support to IPF patients and caregivers who both experienced losses. Therefore, it is necessary to develop palliative care for psychological, social and spiritual distress that is informed by patients' experiences and not only to alleviate symptoms but also to attain the highest quality of life possible. Moreover, introducing multidisciplinary and collaborative care was reported to improve the patient's end‐of‐life care (Rajala et al., 2016) and reduced emergency room visits and hospitalization due to breathing difficulty (Sampson et al., 2015). Support by multidisciplinary teams was thought to bring some emotional stability to the patient because of the increased number of staff attending the patient.

The case study describing patients' panic attacks due to breathing difficulty brought up an important aspect of dyspnoea. Dyspnoea can be negatively influenced by emotional aspects particularly fear. Therefore, the vicious cycle must be interrupted and is a necessary component of care as well as the management of physical symptoms (Kinzel, 1991). Support from multiple disciplines though integrated care including psychological and emotional care can be considered as support for patients and their families, but since it was a retrospective study, prospective studies are needed.

### 5.2 Advanced care planning in palliative care of patients with IPF

Advanced care planning was an issue that was noted by several researchers. Lee et al. (2011) described ACP as a component of care for patients with IPF. Bajwah et al. (2015), Kalluri et al. (2017), Lindell et al. (2017) and Rajala et al. (2016) all reported the necessity for ACP. Furthermore, Ahmadi et al. (2016) included, “end‐of‐life discussion.” The place and timing of the end‐of‐life discussions could begin either during hospitalization or in outpatient situations (Lee, Mira‐Avendano, Ryu, & Daniels, 2014). In addition, Gilbert and Smith (2009) stated that discussing end‐of‐life issues needed to occur earlier in the disease process rather than during a disease crisis. The end‐of‐life discussion is thought to be foundational to the process of ACP. Thickett et al. (2014) supported the British guidelines urging ACP be conducted from the time of diagnosis. Overgaard et al. (2016) explained that ACP is a systematic approach to end‐of‐life conversations that can be used to provide the necessary information for patients and their caregivers, taking into account the patients' values and wishes for the future. Moreover, reflections on life are discussed and a structured approach from the health professional is recommended along with a plan for palliation at an early stage of the disease.

Yet, some patient resistance to ACP has been reported (Lindell et al., 2017) and Lee et al. (2014) also described the existence of stigma against hospice and palliative care. The stigma surrounding palliative care and hospice is evident from the Taiwan study where researchers found that a significant number of public and private facilities do not even use the terms palliative or hospice opting for more hopeful terms like heart, love, grace and peace (Dai, Chen & Lin, 2017). Curtis, Patrick, Caldwell, and Collier (2017) reported that non‐white patients tended to talk about barriers or stigmas as, “I feel that if I talk about death, it could bring death closer” and “I don't like to talk about the care I want if I get very sick.” Also, Igai et al. (2015) conducted questionnaires on end‐of‐life care for nine patients with interstitial pneumonia (IP). 67% of the patients answered, “I do not want to think about it now” in response to the question “Do you want a life extension treatment such as cardiopulmonary resuscitation?” The deeply felt hopelessness and despair may be embedded in the stigma against palliative care, advanced care planning and hospice care (Collins, McLachlan, & Philip, 2017).

Clearly, professionals responsible for end‐of‐life discussions need some support and training to accomplish this most sensitive discussion. Hebert, Moore, and Rooney (2011) reported that the literature includes little description of how nurses learn these advocacy behaviours as advanced care planning and current nursing education lacks information on end‐of‐life care; the barriers to providing high quality end‐of‐life care are common in nursing practice. Making a decision on end‐of‐life care influences the experience of the dying process (Gilbert & Smith, 2009) and the ACP is suggested as helpful in thinking through preliminary instructions (Egan, 2011). This report substantiated that health‐care providers need more training.

Raghu et al. (2011) noted advanced directives and end‐of‐life care issues should be addressed in the ambulatory setting for all patients with IPF, particularly those with severe physiologic impairment and comorbid conditions. Although several researchers suggested starting discussions at the time of diagnosis and early in the disease trajectory, ill effects are often delayed. Therefore, it may be difficult for healthcare providers to begin the ACP discussions with patients because although IPF is an intractable disease, it is not a malignancy nor is it well known like cancer. Predicting the prognosis of IPF is difficult; therefore, healthcare providers may think that ACP can wait. However, patients with IPF can have an acute exacerbation and it can end in respiratory failure. Most patients do not know anything about IPF when they were diagnosed and therefore would not request ACP. A healthcare provider, well‐trained in ACP, would know how to begin the discussion even in the early stages of the disease.

In Japan, it is reported that patients with IPF have 40% more deaths due to acute exacerbations and was greater than that of respiratory failure (Natsuizaka et al., 2014). Researchers found that families made the decision for resuscitation at the time when the patient suddenly experienced an acute episode (Arita et al., 2010). Therefore, the process of ACP is important in reflecting the intention of the patient receiving medical care. The ACP is a process of thinking how patients want to live their lives. Although there is a report of resistance to ACP, the ACP is ultimately helping patients and medical staff to think about their future way of living and the thought is that this provides the patient with a process to control their own future.

For patients with severe COPD, advanced directives and good advanced care planning are thought to provide opportunities to improve the quality of palliative care (Curtis, 2008). Making a decision on end‐of‐life care influences the experience of the process of death (Gilbert & Smith, 2009) and the ACP is suggested to be helpful in thinking through preliminary instructions (Egan, 2011).

Au et al. (2012) reported that an intervention using patient‐specific feedback about preferences for end‐of‐life care and communication between patients with COPD and their clinicians improved the occurrence and quality of communication from patients' perspectives. Bekelman et al. (2017) developed a guide that included questions to: (a) elicit patient understanding of and attitudes toward the future of their illness; (b) clarify values and goals; (c) identify end‐of‐life preferences; and (d) agree on a follow‐up plan. They reported that the structured “goals of care communication guide” were iteratively designed, implemented by nurses and social workers and were feasible based on administration time and acceptability by patients and providers. Using such a guideline for ACP is helpful not only for patients with IPF and their carers but also for healthcare providers as they deal with complex end‐of‐life issues and the stigma surrounding palliative or hospice care (Collins, et al., 2017).

### 5.3 Limitations

There were several limitations that should be addressed. It was possible that despite efforts to locate all relevant extant studies, some were missed. The language restriction to English and Japanese was also a limitation. Regardless of these potential limitations, the findings were fairly consistent regarding the desire and need for early palliative care.

## 6 CONCLUSION

Narrative review of the literature in palliative care of patients with IPF included 19 articles. The majority were qualitative and retrospective studies. Moreover, the qualitative studies described phenomena experienced by patients with IPF. Two intervention studies were carried out that focused on integrated care. Mixed methods research added to a deeper understanding of patients' experiences with IPF. However, there were no studies of advanced care planning for patients with IPF.

## 7 RELEVANCE TO CLINICAL PRACTICE

The palliative care system for patients with IPF was weak and the development of palliative care for patients with IPF was limited. Palliative care for patients with IPF needs to include interventions based on their illness specific experiences.

## CONFLICT OF INTEREST

The author declares that they have no conflict of interests.
