# Supplementary material for: A narrative literature review of palliative care regarding patients with idiopathic pulmonary fibrosis
Source: Nurs Open. 2018 Jun 3;5(4):536–45. doi: 10.1002/nop2.163 (PMC6177544; doi:10.1002/nop2.163)
Supplement: Supplementary file 1 [file NOP2-5-536-s001.docx]

What does this paper contribute to the wider global clinical community?

・This paper contribute to understand to need palliative care of early time to maintain the quality of life of patients through self-care with a stable emotion for patient with idiopathic pulmonary fibrosis.

・This paper contribute to establish structures for receiving palliative care that takes into account the coordination of lifestyle changes from the time of disease diagnosis for patient with idiopathic pulmonary fibrosis.

・This paper contribute to develop palliative care programs to help relieve symptoms and provide psychological and emotional support to patients for idiopathic pulmonary fibrosis living with illness.
